# Common variants explain a large fraction of the variability in the liability to psoriasis in a Han Chinese population

**DOI:** 10.1186/1471-2164-15-87

**Published:** 2014-01-30

**Authors:** Xianyong Yin, Nathan E Wineinger, Hui Cheng, Yong Cui, Fusheng Zhou, Xianbo Zuo, Xiaodong Zheng, Sen Yang, Nicholas J Schork, Xuejun Zhang

**Affiliations:** 1 Institute of Dermatology, Department of Dermatology, The First Affiliated Hospital, Anhui Medical University, Hefei, Anhui Province 230032, China; 2 Key lab of Dermatology, Ministry of Education, State Key Lab of Dermatology Incubation Center, Anhui Medical University, Hefei, Anhui Province 230032, China; 3 Key Lab of Gene Resource Utilization for Complex Diseases, Hefei, Anhui Province 230032, China; 4 Collaborative Innovation Center for Complex and Severe Dermatosis, Anhui Medical University, Hefei, Anhui Province 230032, China; 5 Scripps Health, La Jolla, CA 92037, USA; 6 The Scripps Translational Science Institute, La Jolla, CA 92037, USA; 7 Department of Molecular and Experimental Medicine, The Scripps Research Institute, La Jolla, CA 92037, USA

**Keywords:** Psoriasis, Polygenic, Genome-wide association study, Heritability

## Abstract

**Background:**

Psoriasis is a common inflammatory skin disease with a known genetic component. Our previously published psoriasis genome-wide association study identified dozens of novel susceptibility loci in Han Chinese. However, these markers explained only a small fraction of the estimated heritable component of psoriasis. To better understand the unknown yet likely polygenic architecture in psoriasis, we applied a linear mixed model to quantify the variation in the liability to psoriasis explained by common genetic markers (minor allele frequency > 0.01) in a Han Chinese population.

**Results:**

We explored the polygenic genetic architecture of psoriasis using genome-wide association data from 2,271 Han Chinese individuals. We estimated that 34.9% (s.e. = 6.0%, P = 9 × 10^-9^) of the variation in the liability to psoriasis is captured by common genotyped and imputed variants. We discuss these results in the context of the strong association between HLA variants and psoriasis. We also show that the variance explained by each chromosome is linearly correlated to its length (R^2^ = 0.27, P=0.01), and quantify the impact of a polygenic effect on the prediction and diagnosis of psoriasis.

**Conclusions:**

Our results suggest that psoriasis has a substantial polygenic component, which not only has implications for the development of genetic diagnostics and prognostics for psoriasis, but also suggests that more individual variants contributing to psoriasis may be detected if sample sizes in future association studies are increased.

## Background

Genome-wide association studies (GWAS) have achieved a good deal of success in establishing thousands of individual SNPs (single nucleotide polymorphisms) associated with various common diseases and complex traits (http://www.genome.gov/gwastudies/). However, the SNPs identified in these studies collectively explain only a small fraction of the heritable components of these traits as estimated from twin studies, leading the research community to question where the remaining heritable component of these traits resides – a phenomenon and quest commonly referred to as the search for sources of ‘missing heritability’ [1]. Past work has suggested that a considerable amount of missing heritability for common diseases and complex traits can be accounted for by factors not easily detectable with standard GWAS analysis techniques, such as the contribution of rare variants, common variants with weak individual effects, or a combination of the two [2]. In fact, researchers have explored data analysis techniques designed for the express purpose of detecting a polygenic effect based on the assumption that common variants with small effects contribute to common traits [3]. While the effects of the individual variants may be too small to detect using traditional GWAS approaches, the collective or polygenic effect of numerous markers can be pronounced enough to be detected through these analyses [3-5].

Psoriasis is a common inflammatory skin disease which affects between 0.1 to 5.0% of the population worldwide based on geographic region [6]. In total, more than 40 susceptibility genes have been identified through GWAS involving diverse ethnic populations [6-13]. Across these studies, variants with large effects in the HLA region are consistently shown to be associated with psoriasis [14]. We have previously published the first psoriasis GWAS in a Han Chinese population where we identified 11 novel susceptibility loci [6,11,13]. Consistent with prior evidence that suggests GWAS identified SNPs have only explained roughly 20% of the estimated heritability [12], the variants we identified did not markedly reduce the unexplained heritability. Therefore, in the present study we directly investigated the polygenic architecture of psoriasis in the Han Chinese population. We also set out to determine the degree to which this polygenic component contributed to psoriasis susceptibility over-and-above variants in the HLA region. We leveraged available data analysis tools, most notably the program GCTA which implements a linear mixed model to characterize polygenic effects [5], to estimate the proportion of variation in liability to psoriasis that can be captured by the collective effect of common genome-wide markers (i.e., a polygenic effect) in a Han Chinese sample. We also considered the degree to which markers on each chromosome contributed to psoriasis susceptibility [5,15] as well as the ability to differentiate psoriasis cases and controls on a purely genetic basis if, or when, all markers contributing to this lower bound on heritability are identified.

## Methods

### Participant samples

Our study includes 1,139 psoriasis cases and 1,132 healthy controls from the Han Chinese population. Cases were positively diagnosed by at least by two dermatologists and controls had no psoriasis, no familial history of psoriasis, and no other forms of autoimmune diseases. Written informed consent was provided by all participants. This study was approved by the institutional review committee of the First Affiliated Hospital, Anhui Medical University, China, according to the Declaration of Helsinki.

### Genotyping, imputation and quality control

The samples were genotyped on the Illumina Human610 Quad BeadChip human array as described previously [13]. Samples were excluded which had call rates less than 0.9 per sample per SNP. Marker preparation and analytical implementation for imputation were performed as follows: genetic markers were excluded which demonstrated high missingness (> 0.05), failed Hardy-Weinberg equilibrium (P < 0.0005), or had exceedingly rare alternative alleles (minor allele frequency < 0.005). The remaining genetic data were pre-phased, and genome-wide imputation was performed on the resulting haplotypes using the default parameters in IMPUTE v2.2.2 [16]. The 1000 Genomes Phase 1 integrated variant set haplotypes were used as the reference panel [17]. Genomes were divided into approximately 5 Mb segments (avoiding chromosome and centromere boundaries) with phasing and imputation calculated on each. Imputed markers with information values less than 0.5 were removed from the analysis. GTOOL v0.7.0 was used to convert imputed genotyped posterior probabilities into calls. Genotypes were considered missing if the posterior probability of any genotype was not greater than 90%. In both genotyped and imputed datasets, identical quality control procedures were applied resulting in the exclusion of markers with minor allele frequency < 0.01; call rate < 0.9; and deviation from Hardy-Weinberg equilibrium in the controls (P < 10^-6^).

### Polygenic inheritance analysis

A genetic similarity matrix was constructed based on published methods [16]. Subjects were excluded such that all pairs had estimated genetic relationship less than 0.025. This resulted in the exclusion of 11 and 20 samples in the genotyped and genotyped and imputed datasets, respectively. The proportion of the liability in phenotypic variance explained by genetic markers was calculated using a linear mixed model, implementing restricted maximum likelihood (REML) analysis. Two kinds of analyses (which we refer to as ‘Separate’ analysis and ‘Joint’ analysis) were explored in chromosomal and minor allele frequency partitioning. In the separate analysis, the genetic relationship matrix was estimated separately for 22 individual chromosomes and each allele frequency partition. For the joint analysis, the genetic relationship matrix was built simultaneously across all chromosomes and SNPs with diverse MAFs. We considered the prevalence of psoriasis in Chinese Han population at 0.47% per previous reports [18]. Our analyses were performed using the GCTA software [5]. In all analyses the top 20 principal components were included as covariates to control for potential population stratification. Previously identified genome-wide significant loci were established through literature review. The start and end position for each locus was identified according to dbSNP 130. Overlapping regions were merged.

## Results

In total, 494,641 genotyped and 5,610,687 imputed autosomal SNPs passed quality control thresholds (see Methods). We refer to the genotyped data as set G and genotyped and imputed data as set G + I (Table 1). Since polygenic analyses can be framed in a number of different contexts, we briefly consider our results in terms of the total variation attributable to the collective effect of common variants, the variation attributable to common variants on each chromosome, an assessment of the contribution of lower frequency variants to a polygenic component, and an assessment of the implications of a polygenic effect on the diagnosis of psoriasis.

**Table 1:** The comparative result of phenotypic variance explained by common variants

**Type of data**	**No. of autosomal SNPs**	**No. of cases**	**No. of controls**	**h**^**2**^_**SNP**_**(s.e.)**	**p value**
G	494,641	1137	1123	33.2% (7.0%)	3 × 10^-6^
G + I	5,610,687	1135	1116	34.9% (6.0%)	8 × 10^-9^
HLA region in G + I	33,190	1135	1116	13.2% (2.0%)	<10^-9^
Reported loci in G + I	77,919	1135	1116	14.1% (1.0%)	<10^-9^

h^2^_SNP_: Proportion of Variance in liability to psoriasis vulgaris explained by autosomal SNPs. s.e.: standard error.

Type of Data: G: All Genotyped; G + I: All Genotyped and Imputed; HLA region in G + I : All Genotyped and Imputed data in HLA region; Reported loci in G + I: 12 Reported loci data in Genotyped and Imputed data.

### Genomic variation captured by common SNPs

In the G dataset, we estimated 33.2% of variation in liability to psoriasis (h^2^_SNP_) was explained by all autosomal SNPs (s.e. = 7.0%, P = 3 × 10^-6^). This value rose slightly to 34.9% (s.e. = 6.0%, P = 9 × 10^-9^) in the G + I dataset (Table 1). In the G + I dataset, we extracted SNPs in the HLA region (chr6: 29,700 kb-33,300 kb, including 33,190 SNPs), and found that 13.2% (s.e. = 2.0%, P < 10^-9^) of phenotypic variance was explained by HLA markers (Table 1). We then extracted SNPs from 11 other previously identified susceptibility regions in addition to the HLA region. Based on genetic similarity quantified by variants at these 12 loci (77,919 SNPs), we estimated h^2^_SNP_ to be 14.1% (s.e. = 1.0%, P < 10^-9^) (Table 1).

### Partition of genomic variation by chromosome

**Figure 1:**
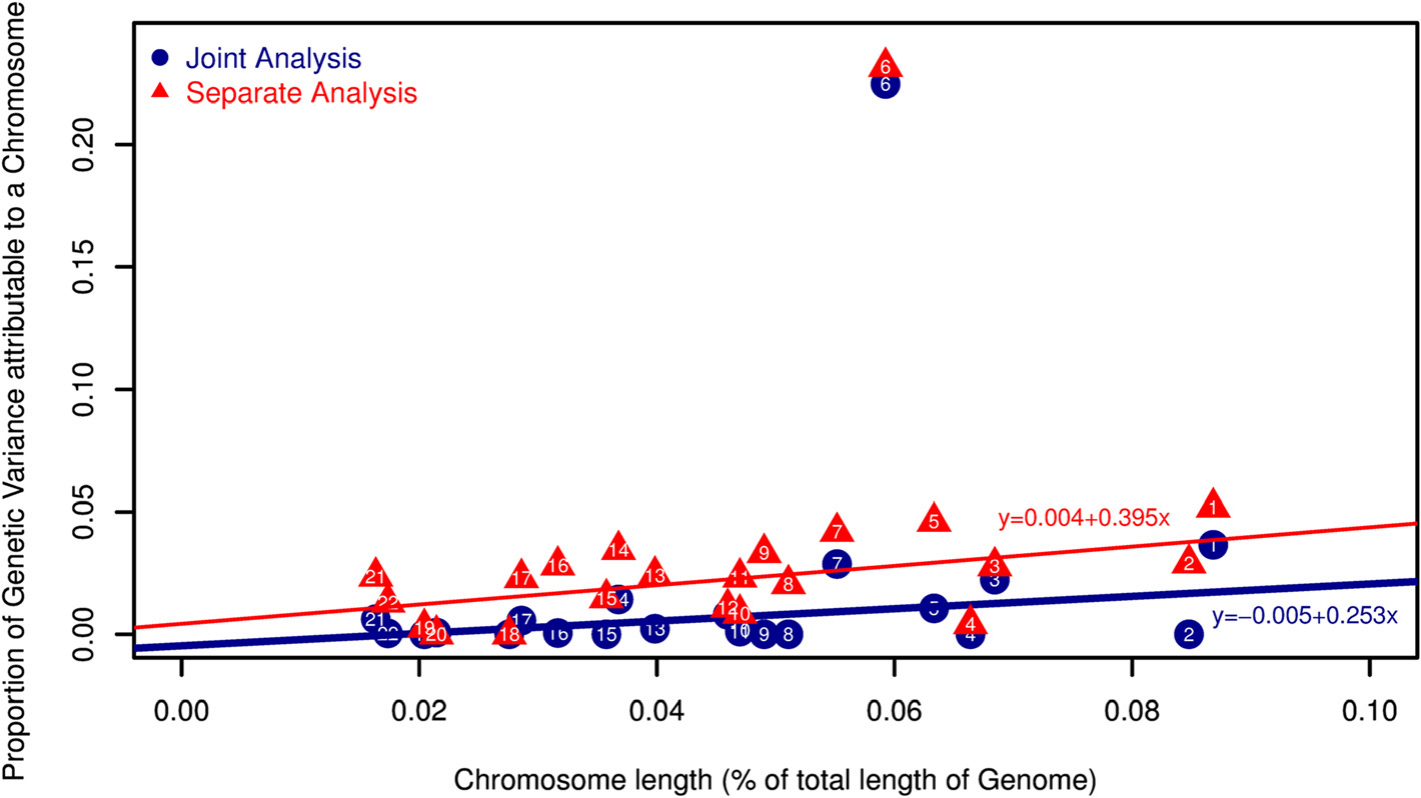
The proportion of liability explained by markers on each chromosome.

We partitioned the genomic variation explained a polygenic effect of common variants by chromosome in the G + I dataset through two kinds of analyses. The first, the ‘separate analysis’ was pursued by fitting a genetic similarity matrix separately for each autosomal chromosome [5]. For the second analysis, the ‘joint analysis’, the genetic similarity matrices were fit simultaneously for all 22 autosomal chromosomes. We observed a positive linear correlation between the estimates of variance explained by each chromosome and the relative length of the chromosome in both analyses (R^2^_sep_ = 0.27, P_sep_ = 0.01; R^2^_joint_ = 0.21, P_joint_ = 0.02) after omitting chromosome 6 due to its exceptional contribution (Figure 1). This observed correlation was consistent with a polygenic effect that has been detected for other traits and diseases [19,20]. In addition, since the estimates obtained from the separate and joint analysis were consistent, we were confident that the relationship between chromosomal length and percent variation in psoriasis liability explained were robust. We note that the largest proportion of variation in liability to psoriasis was explained by the HLA region on chromosome 6 for both the separate and joint analysis approaches.

### Partition of genomic variation by minor allele frequency

**Figure 2:**
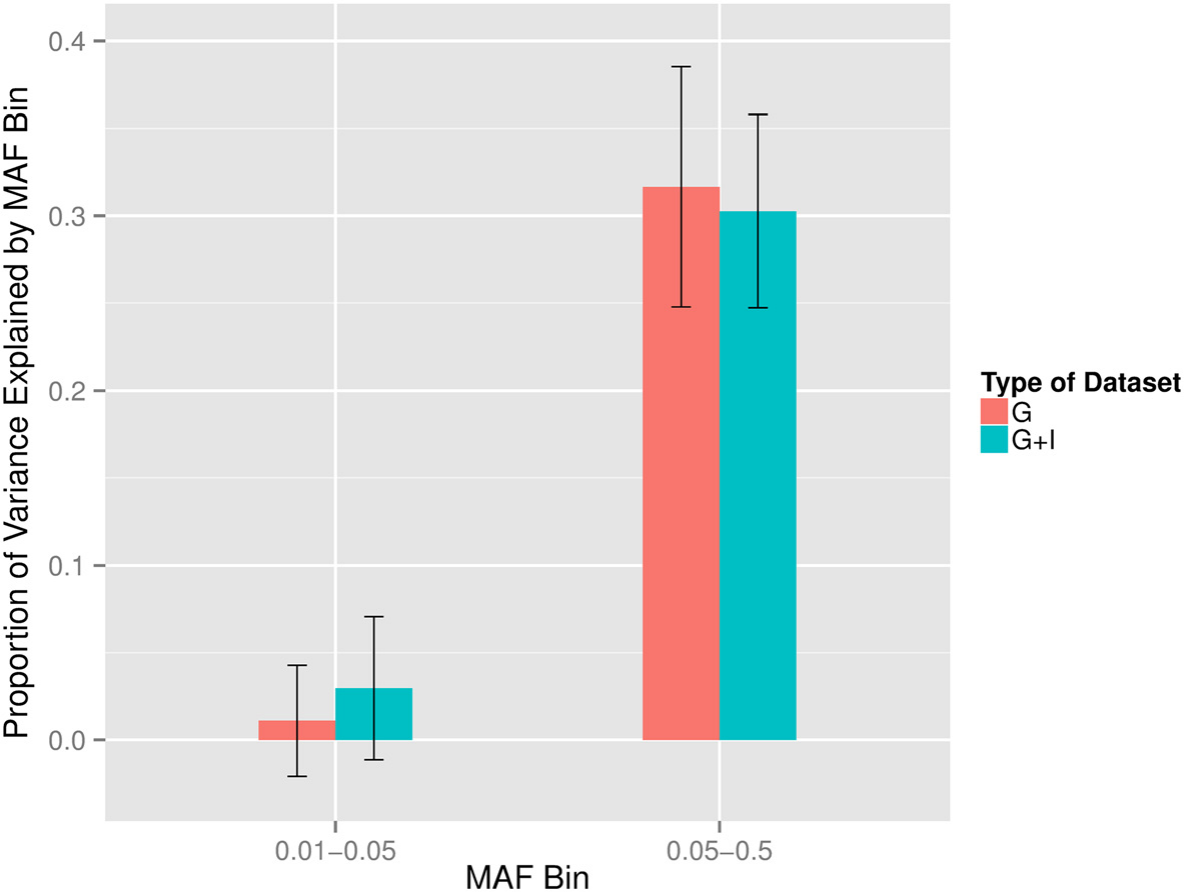
The proportion of liability explained by marker based on minor allele frequency.

We also partitioned the variation attributable to a polygenic effect captured by common variants into two components defined by SNP minor allele frequency (MAF): frequent (MAF > 0.05) or infrequent (0.01 < MAF < 0.05). Markers with infrequent variants in the G and G + I datasets explained 1.1% (45,769 SNPs, s.e. = 3.0%) and 3.0% (1,235,720 SNPs, s.e. = 4.0%) of the variation in liability to psoriasis, respectively. Markers with frequent variants captured greater than 30% of the phenotypic variation in this population in both datasets (Figure 2). It should be noted, however, that although imputation procedures improved the coverage of infrequent variants, the proportion of uncommon SNPs was still only 9.3% and 22% in the G and G + I datasets, respectively. Thus, any conclusive inference on the overall contribution of variants based on minor allele frequency should be qualified by the limitations of our study with respect to sample size and our reliance on genotyping chips and imputation strategies.

### Polygenicity and diagnostic potential

Given our estimates of h^2^_SNP_, we were interested in determining how well psoriasis cases and controls could be distinguished on a purely genetic basis if all common markers which contribute to h^2^_SNP_ were identified [21,22]. These analyses give an indication of how useful our understanding of the genetic basis of psoriasis may be in terms of genetically diagnosing the disease based on GWAS data. These analyses also require assumptions about the overall prevalence of psoriasis, so we considered a few different, yet realistic assumptions about the prevalence (i.e., 0.1 to 0.6%), as well as the overall heritability of psoriasis attributable a polygenic component based on our h^2^_SNP_ estimate of roughly 35% and its standard error (6.0%). Using the *genroc* calculator [21] (available at: http://gump.qimr.edu.au/genroc/) and assuming the prevalence of psoriasis among Han Chinese is 0.47% [18], a polygenic predictor that explains 35% of the variation in liability to psoriasis will have an area under the receiver operating characteristic curve (AUC) value of 0.91. This suggests that there is a 0.91 probability of classifying a random individual with psoriasis correctly relative to a random individual without psoriasis. We varied both the prevalence (0.1-0.6%) and the estimate of heritable component of psoriasis attributable to a polygenic effect (27-41%) and found that the AUC remained consistently high (0.88-0.96), implying that genome-wide commonly genotyped markers may one day be used as a robust statistical classifier for psoriasis diagnosis.

## Discussion

In this study we sought to shed light on the polygenic basis of psoriasis in the Han Chinese population. Our results suggest that more than one-third of variation in liability to psoriasis could be captured by the collective effect of common SNPs. Combined with our previous findings in GWAS, we conclude that a substantial portion of heritability is ‘hidden’ from standard single locus-oriented analysis techniques in our population. This suggests, however, that large-scale GWAS efforts may have potential to recover additional common variants associated with susceptibility to psoriasis, but will likely require much larger sample sizes due to the small effect sizes of these loci. We also find evidence that the proportion of variation explained by individual chromosomes is positively correlated with chromosomal length, which is also consistent with the notion that susceptibility of psoriasis has a polygenic basis. In addition, we found that uncommon variants that were genotyped or imputed did not substantially contribute to our estimate of h^2^_SNP_. Finally, we have shown that the substantial polygenic basis of psoriasis has the potential to accommodate genetic diagnoses of psoriasis.

Although there has been continued debate as to whether the heritability of common diseases is mostly accounted for by common variants with small effects or rare variants with larger effects, there have been few definitive studies to settle this debate. Our study demonstrates that common SNPs are likely to explain a large portion of the heritability in psoriasis among Han Chinese. Given that the reported loci in our previous GWAS explain a modest fraction of the heritability estimated from twin studies and our estimate of h^2^_SNP_ in the present study, we believe that other common variants which did not meet the significance threshold remain to be identified. Data from previous studies have shown that markers identified through GWAS explain 14.3% of the total variation in psoriasis risk. Based on calculation, a classifier which is able to explain 15% of the variation in psoriasis risk will have an AUC of 0.8. Thus, a purely genetic diagnosis of psoriasis may be within reach. However, large sample sizes (N > 50,000) will be required to detect additional markers with increasingly small effects (odds ratios < 1.1) [12,23,24].

To further illustrate the important role of the collective effect of common variants each with small effect on psoriasis, we implemented a partitioning analysis based on minor allele frequency in our datasets. We find that only a small fraction of phenotypic variation can be attributed to SNPs with low frequent variants*.* However, this may be due to the under-representation of SNPs with low minor allele frequency on the genotyping array and imputation strategies we used. In addition, it is expected that weaker additive genetic effects are not well tagged by SNPs with low frequency due to weak linkage disequilibrium with rare causal variants. Our estimate of h^2^_SNP_ was less than half of established heritability of psoriasis in the Han Chinese population [25]. This difference can partially be attributed to imperfect linkage disequilibrium between causal and genotyped variants, imperfectly imputed variants, and genetic interactions, in addition to other sources – but also notably rare variants. Thus, rare variants which are not well captured by our approach may contribute to this difference.

We find that more than 13.0% of the liability to psoriasis can be explained by markers in the HLA region alone, which is consistent with overwhelming evidence from GWAS studies implicating variants in this region. It is notable that chromosome 6 explained a large proportion of phenotypic variation. Although this may be a result of the high linkage disequilibrium between variants in the HLA region, this nevertheless highlights the important role of the HLA locus in the susceptibility of psoriasis. In addition, although several specific susceptibility genes and variants in the HLA region have been revealed [14], future studies using sequencing approaches may be needed to identify actual causal genes and variants in this region. This may be important in further improving a genetic-based diagnostic for psoriasis. Also, given that there may be non-genetic factors contributing to psoriasis, the inclusion of these factors in a psoriasis diagnostic would likely improve its reliability and utility. Thus, despite the fact that psoriasis seems to have a large polygenic component that may make it difficult to identify each variant contributing to disease, there is potential for a genetic diagnosis of using whole genome genotyping and analyses.

## Conclusions

We have performed a polygenic analysis of psoriasis in Han Chinese samples. We estimated the contribution of common variants to psoriasis phenotypic variation. Our study suggests that substantial polygenic component has been hidden in psoriasis, which not only has implications for the development of genetic diagnostics and prognostics for psoriasis, but also suggests that more individual variants contributing to psoriasis may be detected if sample sizes in future association studies are increased.

## Competing interests

The authors declare that they have no competing interests.

## Authors’ contributions

XJZ and NJS designed this study, analysis and interpretation of results and helped to revise the manuscript. XYY conducted the analysis as well as wrote the manuscript. NEW conducted the imputation analysis and participated in the revising the manuscript. SY, HC, YC collected the samples. FSZ, XBZ and XDZ conducted the genotyping experiments and genotype calling. All authors read and approved the final manuscript.
